# Effect of acetic acid inactivation of SARS-CoV-2

**DOI:** 10.1371/journal.pone.0276578

**Published:** 2023-02-08

**Authors:** Narayanappa Amruta, Nicholas J. Maness, Timothy E. Gressett, Yoshihiro Tsuchiya, Mikiya Kishi, Gregory Bix

**Affiliations:** 1 Department of Neurosurgery, Clinical Neuroscience Research Center, Tulane University School of Medicine, New Orleans, LA, United States of America; 2 Tulane National Primate Research Center, Tulane University, Covington, LA, United States of America; 3 Department of Microbiology and Immunology, Tulane University School of Medicine, New Orleans, LA, United States of America; 4 Tulane Brain Institute, Tulane University, New Orleans, LA, United States of America; 5 Central Research Institute, Mizkan Holdings Co., Ltd. Aichi, Japan; 6 Department of Neurology, Tulane University School of Medicine, New Orleans, LA, United States of America; University of South Dakota, UNITED STATES

## Abstract

Effective measures are needed to prevent the spread and infectivity of SARS-CoV-2 that causes COVID-19. Chemical inactivation may help to prevent the spread and transmission of this and other viruses. Hence, we tested the SARS-CoV-2 antiviral activity of acetic acid, the main component of vinegar, *in vitro*. Inactivation and binding assays suggest that acetic acid is virucidal. We found that 6% acetic acid, a concentration typically found in white distilled vinegar, effectively inactivated SARS-CoV-2 after 15-min incubation with a complete loss of replication of competent virus as measured by TCID50. Transmission electron microscopy further demonstrated that 6% acetic acid disrupts SARS-CoV-2 virion structure. In addition, 6% acetic acid significantly inhibits and disrupts the binding of SARS-CoV-2 spike protein binding to ACE2, the primary SARS-CoV-2 cell receptor, after contact with spike protein for 5, 10, 30 and 60 minutes incubation. Taken together, our findings demonstrate that acetic acid possesses inactivating activity against SARS-CoV-2 and may represent a safe alternative to commonly used chemical disinfectants to effectively control the spread of SARS-CoV-2.

## Introduction

The human coronavirus disease (COVID-19) caused by severe acute respiratory syndrome coronavirus 2 (SARS-CoV-2) is a respiratory pathogen that emerged in late 2019 [1,2]. As of February 21st, 2022, there were 5,892,174 deaths with a total of 425,430,279 confirmed COVID-19 cases worldwide [3,4]. SARS-CoV-2 can cause acute respiratory distress syndrome (ARDS) due to severe pneumonia and lead to death [5,6]. Additional symptoms include myalgia, diarrhea, and constipation at the onset of illness. SARS-CoV-2 can also cause multi-organ disease and hypercoagulation [7]. In most cases, viral replication occurs in the upper respiratory epithelia via binding to angiotensin-converting enzyme 2 (ACE2), which increases immune responses by cytokine storm [8–10]. Even COVID-19 patients with mild or no symptoms may have high viral loads and be highly contagious, which has greatly worsened the pandemic [6]. COVID-19 has a common characteristic of transmission from infected to non-infected individuals by direct spread via respiratory droplets when sneezing or coughing [11], as well as indirect virus transmission through contact with contaminated surfaces [12–14]. Therefore, it is imperative to utilize effective and cost-efficient means to inactivate high frequency contact and other potentially contaminated surfaces to prevent the spread of infection.

Acetic acid, the active component of vinegar, has been used as a disinfectant for thousands of years to eliminate bacteria from fresh products [15] and is an effective disinfectant against mycobacterial infection [16]. Furthermore, previous studies have reported anti-viral activities of acetic acid [17–19]. In our study, we used white distilled vinegar (acetic acid concentration 6%) as a hand spray to show virucidal activity to inactivate SARS-CoV [17]. Acetic acid causes inactivation and disaggregation of glycoproteins found on influenza viruses that destroys the viral envelope and inhibits viral transmission [18,20]. Recently, it has also been shown to be an adjunctive therapy for non-severe COVID-19 [20] as both Grain Flavored Distilled Vinegar (grain vinegar, GV; 4% acetic acid concentration) and white distilled vinegar have virucidal effects on SARS-CoV-2 [21,22]. Although acetic acid has been shown to have virucidal effects on SARS-CoV-2, the mechanism remains unclear. Therefore, we examined the mechanism.

## Materials and methods

### Cells and virus

Vero E6 (ATCC CRL-1586) cells were cultured with Dulbecco’s modified Eagle medium (DMEM) supplemented with 10% heat-inactivated fetal bovine serum for maintenance of the culture. A stock of SARS-CoV-2 (SARS-CoV-2; 2019-nCoV/USA-WA1/2020 (BEI# NR-52281) was generated by infecting sub-confluent monolayers of VeroE6 cells at an MOI of approximately 0.001 for one hour with minimal volume in serum-free DMEM with rocking every 15 minutes. After one hour, the inoculum was removed and replaced by complete DMEM containing 2% fetal bovine serum. The virus was allowed to propagate at 37°C in 5% CO2. The supernatant was harvested between 48 and 72 hours when cytopathic effect (CPE) was noted in approximately half of the monolayer. The virus was then harvested by clearing the supernatant at 1,000g for 15 min, aliquoting and freezing it at -80C. Stock titer was determined by TCID50 and sequencing was used to confirm that the consensus sequence was unchanged from the original isolate.

### Acetic acid

Acetic acid was purchased from Fisher Scientific (Waltham, MA). Different concentration of Acetic acid stock solutions (6% v/v) were prepared in distilled water from glacial Acetic acid (>99.7% solution) by dissolving acetic acid in distilled water.

### Reagents

Enzyme-linked immunosorbent assay (ELISA) reagents used in study were obtained through BEI Resources, NIAID, NIH: Spike Glycoprotein Receptor Binding Domain (RBD) from SARS-Related Coronavirus 2, with C-Terminal Histidine Tag, Recombinant from HEK293 Cells (NR-52366), and CoV-ACE2S2-1 ELISA kit (Ray biotech, GA, USA) [23,24].

## SARS-CoV-2 neutralization assay

A modified TCID50 assay was used to assess the ability of white distilled vinegar (6% acetic acid in water) to neutralize SARS-CoV-2. Briefly, 100ul of a 1x10^6 TCID50/ml stock (1x10^5 TCID50 total virus) of SARS-CoV-2 (WA1/2020 isolate) was mixed with 6% acetic acid at a ratio of 1:10 (100ul of virus with 900ul of dilute acid) for 15 minutes. After 15 minutes, the mixture was diluted 100-fold with DMEM by combining 250ul of the virus/acid mixture with 24.75ml of DMEM. As a control, this approach was conducted in parallel with virus diluted in water instead of acetic acid. These solutions were overlayed straight and in serial 1:10 dilutions on near-confluent monolayers of VeroE6 cells in 48 well plates. The overlays were incubated at 37°C for 1 hour with rocking every 15 minutes. After one hour, the inoculum was removed and replaced with 1ml of DMEM with 2% FBS and incubated for 14 days and monitored for signs of infection (visual cytopathic effect, CPE) every 2 to 3 days. Plates were scored for infection on day 10 and verified on day 14. The Median Tissue Culture Infectious Dose (TCID50) was calculated using the method of Reed and Muench [25]. The limit of detection for this assay is approximately 200 TCID50/ml.

### Transmission electron microscope (TEM) assay

To visualize acetic acid (or distilled water control) treated SARS-CoV-2 particles, the same conditions were used as with the TCID50 inactivation study but followed by centrifugation of the samples through Amicon Ultra-15 filter devices according to manufacturer (Sigma, St. Louis, MO) recommendations to concentrate particles approximately 10-fold to facilitate visualization. Samples were inactivated by UV in a Biosafety Cabinet (Sterilgard® III advance, Baker, Sanford, Maine) device for 15 minutes, conditions we had validated to neutralize this virus [26]. These samples were used for TEM visualization.

Cryogenic TEM samples were prepared with FEI Vitrobot. A 5 μL drop of sample was loaded on a 200 mesh copper lacey carbon TEM grid, and sufficiently blotted once by filter paper for 2 sec at 100% humidity before the grid was plunged into liquid ethane. After samples were vitrified, they were transferred to and held at -170˚C in a cryogenic TEM holder. Cryogenic TEM images were collected on a FEI TECNAI G2F30 TEM at 200 kV.

## ELISA

This assay was used to determine the ability of acetic acid to inhibit binding of SARS-CoV-2 Spike Receptor Binding Domain (RBD) (SARS-CoV- 2 spike RBD) protein to the ACE2 host cell receptor. For the determination of inhibition of ACE2 binding by vinegar, two different methods were used. In the first method, the SARS-CoV-2 spike protein was immobilized on 96-well ELISA plates (Sigma-Aldrich, Germany) at a concentration of 100 ng/well overnight at 4°C. The coated plates were blocked with 2.5% bovine serum albumin (BSA) for 2 h at room temperature. In separate tubes, the acetic acid was diluted from 1M to 15.625 mM in 1:2 dilutions by using distilled water. The mixture was added to the spike protein-coated plates and incubated for different contact times (5, 10, 30, and 60 minutes) at 37°C. After the respective incubation was completed, acetic acid was then neutralized by assay buffer (10-fold dilution was achieved using assay buffer containing hACE2–Fc (0.5 μg/mL, Cat# 10108-H02H, Sino Biological) and incubated at 37°C for 30 minutes. The plates were washed and incubated with horseradish peroxidase-labeled goat anti-human Fc secondary antibody (SouthernBiotech, Birmingham, AL) at 1:5000 for 30 min at 37°C. The 3,3′,5,5′-Tetramethylbenzidine (TMB) substrate was used as a color developer for colorimetric detection and 2 N H_2_SO_4_ (Sigma-Aldrich, Germany) was added to each well to stop the reaction. Absorbance was read at 450 nm using an ELISA plate reader (Molecular Devices, USA) [27].

In the second method, recombinantly-expressed hACE2 precoated plates (Cat# CoV-ACE2S2-1 ELISA kit, RayBiotech, GA, USA) were used. Acetic acid was diluted from 2M to 250 mM in 1:2 dilutions by using distilled water. The acetic acid-SARS-CoV-2 spike RBD protein concentration (1x) was added into the ACE2 coated wells after the spike proteins were exposed to acetic acid for 30 minutes at 37°C followed by acid neutralization by diluting it 10-fold in assay buffer. The rest of the procedure was as described above and followed the instructions of the manufacturer (Cat# CoV-ACE2S2-1 ELISA kit, RayBiotech, GA, USA).

### Statistics

Data are presented using the mean ± SD. Differences between groups were determined via the 1-way analysis of variance using Dunnett’s post-hoc multiple comparisons test. Data was normalized to no-Acetic Acid vehicle control (stats as compared to respective vehicle). Experiments are represented as the mean ± SD of a total of 3 replicates. For half-maximal inhibitory concentration (IC50) estimation, the data points directly bounding the IC50 value were used, and calculation was made in GraphPad Prism (GraphPad, La Jolla, California). A p value <0.05 was considered statistically significant.

## Results

### Inactivation of live SARS-CoV-2 by 6% acetic acid

**Fig 1:**
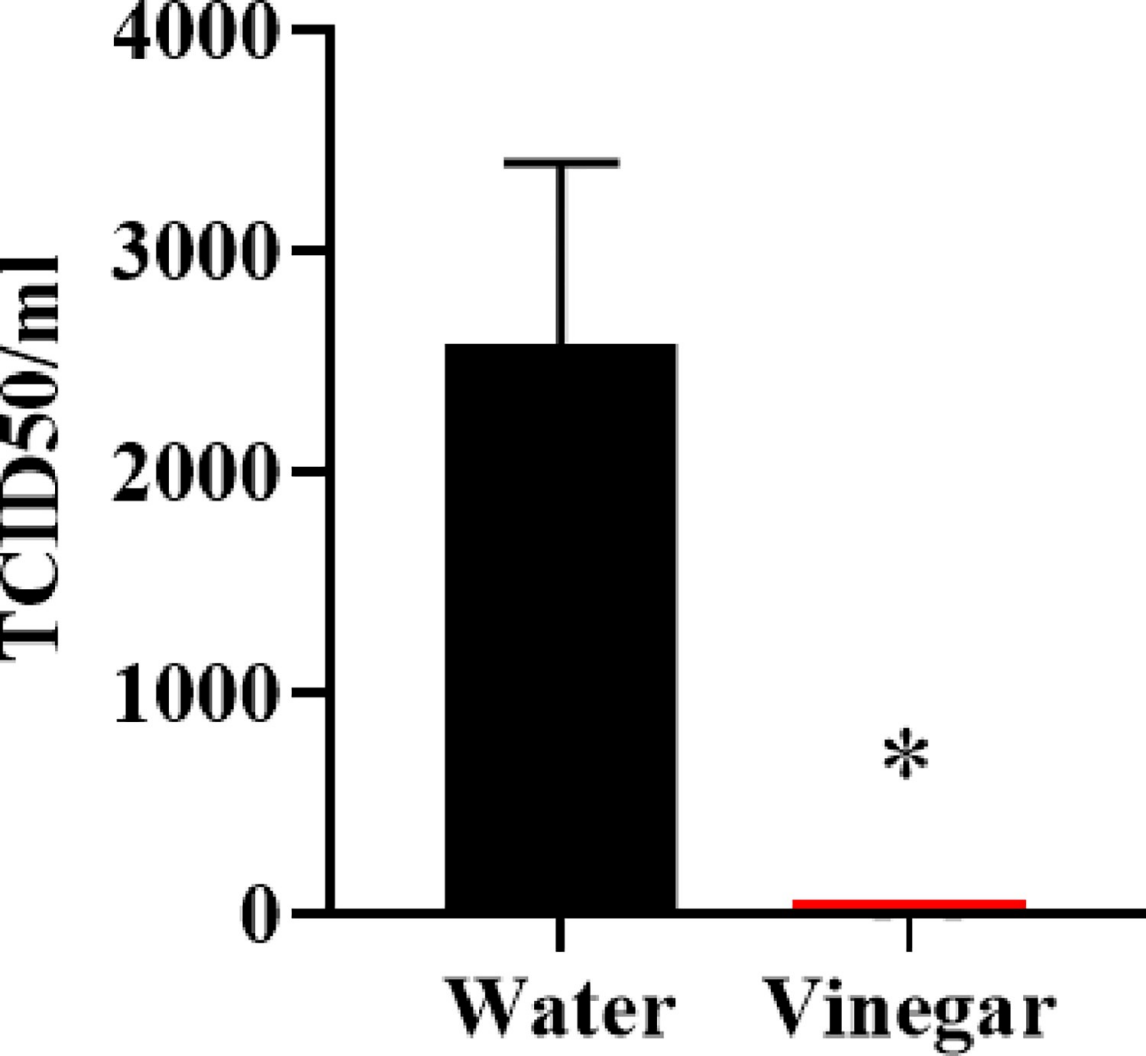
Vinegar (6% acetic acid) inactivates SARS-CoV-2. SARS-CoV-2 (1x10^5 TCID50, WA1/2020 isolate) was incubated with 6% acetic acid (or distilled water negative control) for 15min at a 1:10 ratio. The sample was then diluted 1:100 using water and tested for live virus by TCID50. TCID50 was read 10 days after experimental set up but left in culture for an additional week to ensure that no additional viral outgrowth occurred. Data represent mean ±SD, n = 2 *p < 0.05.

We tested whether acetic acid diluted to 6% to mimic household white distilled vinegar would inactivate SARS-CoV-2. We found that treatment of a 1x10^5 TCID50 of SARS-CoV-2 with 6% acetic acid at a ratio of one part virus solution to 10 parts acetic acid (or distilled water for control) for 15 minutes resulted in a complete loss of replication competent virus as measured by TCID50. Sample datasets used to calculate the 50% endpoint using the Reed–Muench method [25], while live virus remained detectable in the water treated virus (Fig 1).

### Effect of 6% acetic acid on SARS-CoV-2 viral particle morphology by transmission electron microscope (TEM)

**Fig 2:**
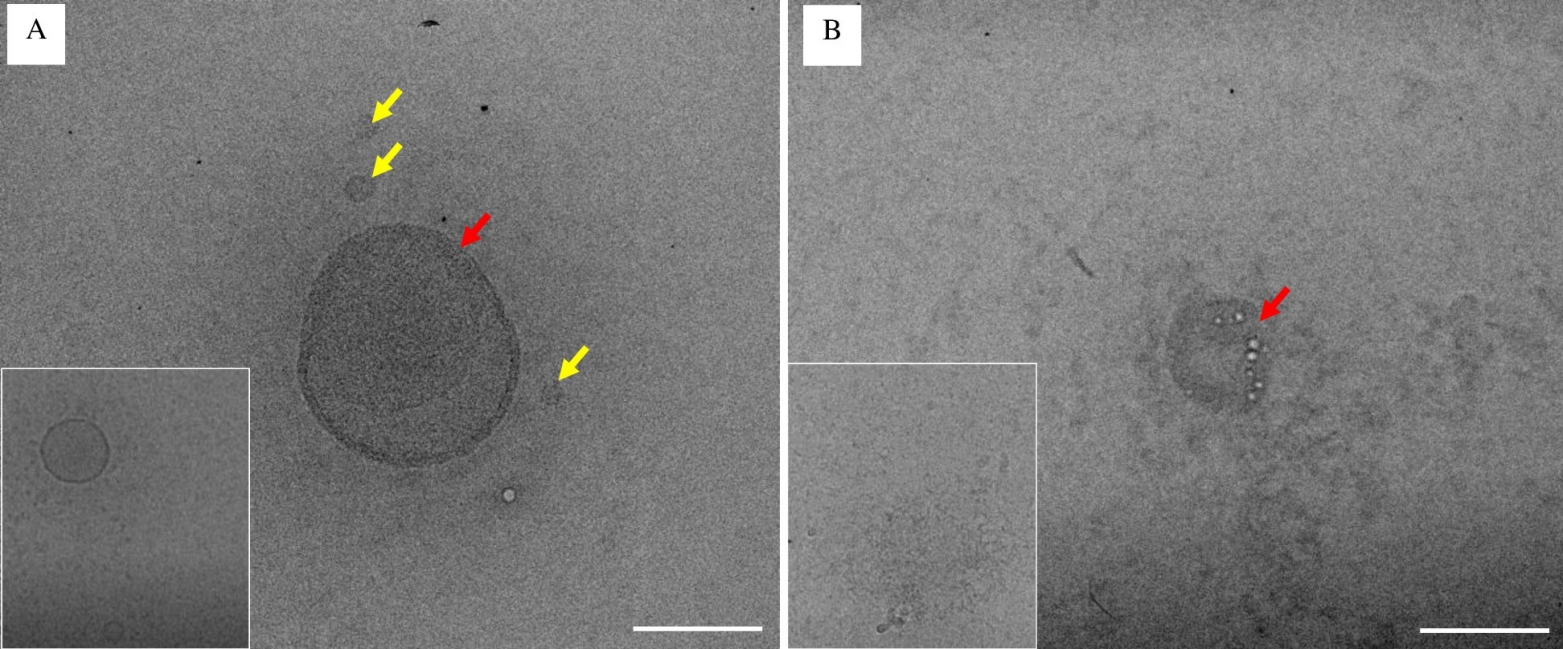
Effect of vinegar on SARS-CoV-2 viral particles by transmission electron microscope (TEM). (A-B) Representative TEM images. (A) The untreated cells, water (- vinegar) SARS-CoV-2 viral particles shows morphodiagnostic features of family *Coronaviridae* with morphologically intact structure whereas (B) Cells treated with 6% acetic acid (+ vinegar) SARS-CoV-2 viral particles showing presence of abnormal viral morphodiagnostic with misshapen structure with fewer viral particles, and disorganized virion structure. Scale bar = A-B 100nm. Insets are shown from additional images of the same sample.

We used TEM microscopy to observe the morphology of SARS-CoV-2 virus virions treated with 6% acetic acid (+ vinegar). The typical morphodiagnostic features of coronavirus are approximately 80 nm in diameter with round structures. The negative-stained virus particles especially virus particle showing typical morphodiagnostic features of coronaviruses. These viruses showed round shape with smaller size (yellow arrow) and some of them had similar morphological features of coronavirus structure with 75 nm in size with distinct envelope projection having peplomeric structures [28,29]. Representative images from the untreated cells, water (- vinegar) SARS-CoV-2 viral particles shows morpho-diagnostic features of family Coronaviridae with morphologically intact structure (Fig 2A, red arrow). Cells treated with 6% acetic acid / vinegar (+ vinegar) SARS-CoV-2 viral particles shows the presence of abnormal viral morpho-diagnostic features with misshapen structures, fewer viral particles in number, and disorganized virion structure (Fig 2B).

### Effects of acetic acid on SARS-CoV-2 spike protein and ACE2 binding

**Fig 3:**
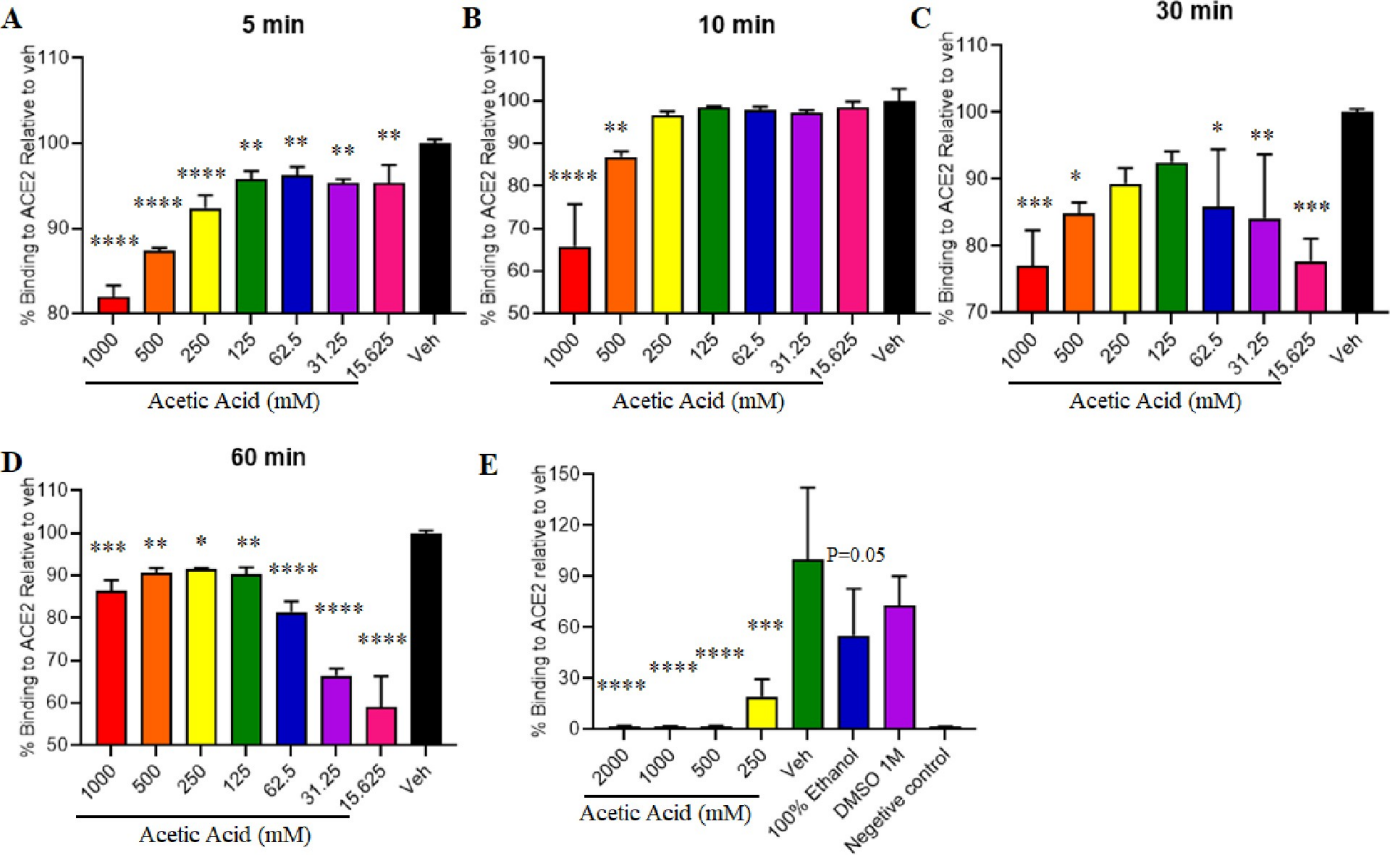
Acetic acid effects on SARS-CoV-2 spike and ACE2 binding. Enzyme-linked immunosorbent assay data indicates that Acetic Acid or Vinegar (Acetic Acid at 1M, or at 2 M) alters binding of Spike to human angiotensin-converting enzyme (ACE2). **(A-D)** When spike-coated plates are incubated with concentrations of Acetic Acid and the effect of Acetic acid was tested when in contact with spike protein for (A) 5 minutes; (B) 10 minutes; (C) 30 minutes; (D) 60 minutes; as well as **(E)** when ACE2 coated plates are incubated with Spike RBD protein and different concentrations of Acetic Acid was tested when in contact with spike protein for 30 minutes. Data was normalized to no-Acetic Acid vehicle control (stats as compared to respective vehicle). Data represent mean ±SD, n = 3 *p < 0.05; **p < 0.01; ***p < 0.001; ****p < 0.0001.

In this study, we explored the binding of the SARS-CoV-2 to human angiotensin-converting enzyme (ACE2) with different concentrations of acetic acid. The effect of acetic acid was tested when in contact with spike protein by using ELISA based assays. The data indicate that 6% acetic acid (1 M) / vinegar, or at 2 M alters binding of spike to ACE2 when the spike-coated plates are incubated with acetic acid **(**Fig 3A–3D). All concentrations (15.25, 31.25, 62.50, 125, 250, 500 and 1000 mM) of acetic acid significantly inhibited the binding when in contact with spike protein for 5 and 60 minutes (Fig 3A and 3D, respectively). There was a significant inhibition of binding with maximum effect of acetic acid at 500 and 1000 mM when the spike protein was exposed to acetic acid for 10 minutes (Fig 1B). Similarly, 30 minutes contact time with acetic acid showed a maximum effect at 15.25, 31.25, 62.5, 500 and 1000 mM acetic acid concentrations (Fig 3C) and inhibited spike-ACE2 binding. Interestingly, we observed that the maximum concentration of acetic acid (2 M), and 6% acetic acid (1 M) significantly inhibited the binding of spike to ACE2 when the hACE2-coated plates are incubated after the spike proteins when in contact with acetic acid for about 30 minutes, and the acid has been neutralized (Fig 3E).

## Discussion

The early detection and decontamination of surfaces is an important step to prevent the spread of infectious diseases. Previous studies have shown that white distilled vinegar or acetic acid aqueous solution have virucidal effect against SARS-CoV-2 [21,22,30]. Based on this previous research, in our present study we further investigated the SARS-CoV-2 virucidal and anti-viral effects of acetic acid and demonstrated that 6% acetic acid / white distilled vinegar possesses antiviral activity against SARS-CoV-2 in VeroE6 African green monkey kidney cells. We have shown that 6% acetic acid has reduced detectable replication competent virus to below the limit of detection as measured by median tissue culture infectious dose (TCID50) (concentration which inhibits 50% of viral replication) as evaluated using Reed–Muench method to calculate 50% endpoint. DMEM was used to dilute the vinegar treated virus while the untreated virus clearly remained infectious [25]. As previously shown the desired concentration of the Acetic acid (4%) in contact with SARS-CoV-2 for 1 and 5 min is effective for the virus and a change in pH doesn’t alter the activity of acetic acid on the virus. By contrast, white distilled vinegar with a 6% acetic acid concentration had a stronger inactivating effect in contact with the virus for 10 s and 1 min [22]. This result further supports the potential role of vinegar as a SARS-CoV-2 inactivation to prevent the spread of infection. Lastly, while others have reported that common household cleaning agents, to include vinegar and its active ingredient acetic acid, may be ineffective at reducing viral infectivity, we note that we have previously showed that dilute acetic acid can inactivate the virus [22,31]. Future studies examining the mechanism of inactivation are thus urgently warranted.

In this study, we show TEM analysis which demonstrates that 6% acetic acid disrupts SARS-CoV-2. These virions had abnormal viral morpho-diagnostic appearance with misshapen structure, fewer viral particles number, and disorganized virion structure, while untreated SARS-CoV-2 viral particles had morpho-diagnostic features of the family Coronaviridae with morphologically intact structure. The untreated virions morphology observed is consistent with previous reports [29,32–34].

We additionally show the binding effect of different concentration of acetic acid solution in contact with SARS-CoV-2 spike for 5 min, 10, 30 and 60 minutes through ELISA based assays. All the concentrations of acetic acid showed a stronger effect with reduced binding of the trimeric spike protein to hACE2. In addition, 6% acetic acid / vinegar solution was more effective even with a relatively short contact time of 5 min and at a maximum contact time for 60 minutes when the spike-coated plates were incubated with acetic acid. Furthermore, application of 6% acetic acid / vinegar significantly reduced the binding of the trimeric spike protein to hACE2 as compared to no-acetic acid vehicle control when hACE2-coated plates are incubated after the spike proteins when in contact with acetic acid and the acid has been neutralized. Our results align with previous studies that report that white distilled vinegar with 6% acetic acid had a strong inactivating effect in contact with the virus for 10 seconds and 1 minute [22]. Future studies which examine the effects of other acids with varying pH to neutralize SARS-CoV-2 and other like-viruses with comparison controls proteins may also yield additional mechanistic insight, and should be urgently considered.

Collectively, our results demonstrate that acetic acid disrupts the structure, ACE2 binding capabilities, and *in vitro* infectivity of SARS-CoV-2 at concentrations found in common house-hold vinegar supporting the use of vinegar as a virucidal agent to inactivate SARS-CoV-2 on surfaces to prevent the spread of infection [27].

## Conclusion

Vinegar is inexpensive, widely available, safe, and has minimal toxicity as compared to other cleaners. This makes it an ideal agent to be used for prevention of binding of spike proteins and destruction of SARS-CoV-2 virion structures as demonstrated in our study. Therefore, the ability of the acetic acid active component of vinegar to inactivate SARS-CoV-2 suggests that it could be an important tool in the fight against COVID-19.

## Supporting information

S1 Appendix Supporting information file for data. (XLSX)
